# Development of CD8 and CD4 Regulatory T cells in the Thymus

**Published:** 2026

**Authors:** Gongyi Zhang, Chuan-Yuan Li

**Affiliations:** 1 Department of Immunology and Genomic Medicine, National Jewish Health, Denver, CO 80206, USA; 2 Department of Immunology and Microbiology, School of Medicine, University of Colorado Anschutz Medical Campus, Aurora, CO 80216, USA; 3 Department of Dermatology, Duke University, USA

**Keywords:** CD8 Treg, CD4 Treg, CD25, IL-15R, TGF-beta, FOXO1, FOXP3, PD-1, CTLA4, IL-10, Thetis cells (TCs)

## Abstract

A study by Badr and colleagues showed that self-reactive CD8 T cells left early from the thymus and became PD-1-positive CD8 T cells in the periphery that are tolerant to self-antigens [1]. This report is reminiscent of an earlier study on self-reactive CD4 T cells eventually becoming CD4 T regulatory cells after negative selection in the thymus with high expression of the inhibitory receptor CTLA4 on the cell surface [2]. Thus, a new picture emerges, showing that self-reactive T cells in the thymus disappear after negative selection and reappear in the periphery with different identities: the regulatory T cells. Interestingly, it seems that medulla dendritic cells (mDCs) and thymus-homing T cells (from circulation) are required to generate peripheral Tregs (pTregs) to build up tolerance against foreign antigens as well as some tissue-specific host antigens. A recent interesting discovery showed that the AIRE-expressing Thetis cell family, which is essential for the formation of pTregs [3], could be tissue-specific cells migrating back from the periphery, similar to previously reported Mimetic cells [4]. Furthermore, CD8 Treg could likely be the same population of tumor-infiltrating CD8 T cells, which is currently termed terminally differentiated exhausted CD8 T cells. Finally, the picture of regulatory T cells, including CD4 Treg and CD8 Treg, could be drawn, accounting for both branches of the T cell family.

## Introduction

A ground-breaking discovery by Kappler and Marrack in the middle of the 1980s revealed how T cells are developed in the thymus, which builds central tolerance in animals: the process of positive and negative selection [5]. However, the origins of the major players of central tolerance, the regulatory T cells, remain unsolved or controversial. Much earlier, Gershon and others found that a group of T cells are major players regulating central tolerance [6]. This group of T cells was finally isolated and characterized as CD25-positive CD4 regulatory T cells (CD4 Tregs) by Sakaguchi and colleagues two and a half decades later [7]. Another paradigm-shifting discovery by Caton’s group showed that self-reactive CD4 T cells in the thymus actually developed into CD4 Tregs [2]. That study, though not well appreciated in the past few decades, is the first to explore the origin of CD4 Tregs. Later, it was found that the transcription factor FOXP3 is essential in controlling the expression of signature markers of CD4 Tregs, such as CD25 and CTLA4, by three groups [8–10]. Interestingly, Chen and colleagues found that the expression of FOXP3 was strictly controlled by TGF-beta signaling [11]. Chen’s group later also found that low-, intermediate-, and high-affinity binding between TCRs and medulla epithelial cells (mTECs) MHC II presenting self-antigens could all lead to the development of CD4 Tregs in the presence of high concentrations of TGF-beta within the medulla zone of the thymus [12]. These results corroborated the early discovery by Caton’s group [2] that CD4 Tregs originate from self-reactive CD4 T cells derived from negative selection by the mTECs. An interesting question emerged: What is the destination of self-reactive CD8 T cells after negative selection in the medulla? Badr and colleagues’ recent discovery showed that these self-reactive CD8 T cells also follow a similar pathway to develop into regulatory-like CD8 T cells [1]. Though not claimed as CD8 Treg cells, they are likely the long-sought-after CD8 Treg family. This paradigm-shifting discovery may thus identify a critical missing piece of the puzzle regarding the origin of thymus-derived Tregs. However, there are still important unanswered questions.

### The Origin of CD8 Treg and CD4 Treg against Self-Antigens

The origin of regulatory T cells in the thymus is a long-standing and hotly debated topic. Caton’s group first revealed that CD4 Tregs originated from self-reactive CD4 T cells after negative selection in the thymus [2], although it was not widely and well accepted in the field. Although it was not openly claimed as CD8 Tregs in the Bar’s report, it at least showed that self-reactive CD8 T cells become unresponsive CD8 T cells in the periphery with the functional properties of typical Tregs [1]. It is also well-evidenced from the report that this group of CD8 T cells originates from double-positive T cells, though the authors claimed that these premature T cells are directly evicted from the positive selection stage in the cortex zone in the thymus without experiencing negative selection in the Medulla zone of the thymus. However, it is likely that negatively selected single-positive CD8 T cells quickly exit the Medulla zone, which makes them hard to detect. This actually is also true for CD4 Tregs from the same report. Marilyn mice that only generate CD4 Tregs of HY antigen in male mice also generate single-positive CD4 T cells in female mice but not in male mice (reference [1], Figure 1D). Besides the transient existence of Tregs in the Medulla zone, another interesting conclusion that could be derived from these observations is that a mouse can select either a CD4 T cell or a CD8 T cell during positive selection for the same T cell receptor (TCR). There do not exist the same TCRs with both CD4-positive and CD8-positive T cells (mutually exclusive), as well demonstrated by Weng’s group [13].

### The Origin of CD8 Treg and CD4 Treg against Foreign Antigens and Rare Host Tissue-Specific Antigens

It is still another hotly debated topic regarding the role of dendritic cells (mDCs) within the medulla zone of the thymus. There are mTECs that present self-antigens through MHC I or MHC II in the medulla zone; why is there a need for mDCs? There are numerous interpretations. These mDCs are likely essential for the development of either CD4 or CD8 Tregs against foreign antigens by presenting foreign antigens as well as some leaking tissue-specific host self-antigens derived from circulation, starting early embryonic development in the mother’s womb and continuing after birth till adulthood. This process is essential for individuals to develop tolerance toward beneficial or non-harmful foreign antigens from the outside world, such as nutrition (food), allergens, commensal microbiota, and medulla-missed self-antigens, etc. A report from Dr. Gretchen Diehl’s group showed that there exists a dominant CX3CR1^+^ DC family presenting antigen from commensal segmented filamentous bacteria (SFB) in the thymus [14]. A recent groundbreaking report showed that a newly characterized AIRE-expressing Thetis cell (TC I-IV) family in local lymph nodes, with different molecular marks compared to traditional dendritic cells, is capable of presenting innocuous foreign antigens and is essential for the formation of peripheral regulatory T cells (pTregs) [3]. It is also found that Thetis cells exist in the thymus via publicly available scRNA-seq datasets of thymic APCs (Chrysothemis, private communications). This novel discovery reminds us of a novel Mimetic population of mTECs discovered not long ago, which presents tissue-specific antigens to T cells within the Medulla zone [4]. Interestingly enough, a long-standing puzzle in the field is the function of the population of homing T cells (which come back to the Medulla zone after egressing out) in the thymus, which was identified in the 1960s but was not well appreciated in the field [15]. Dr. Diel’s report also showed that there exist homing back SFB-specific T cells in the Thymus [14]. Unfortunately, they did not track the final destination of this population. Based on the above analysis in the previous section, it is quite likely that these SFB-specific and possibly tissue-specific homing back T cells in the Thymus must be matched with mDCs, Thetic cells, and Mimetic cells, and eventually develop into Tregs. A step further, we believe that this is a process to develop Tregs against foreign antigens and some tissue-specific antigens, building up the peripheral Treg family (pTreg). Applying antibiotics to the mice, the overall Treg number dropped dramatically [14], providing strong evidence to support our speculation. We believe that antibiotics could eliminate some pTregs against SFB. However, three back-to-back reports showed that pTregs are developed in the local draining lymph nodes via novel antigen-presenting cells such as type 3 innate lymphoid cells (ILC3) or Janus cells (JC) [16–18]. If simultaneously investigating what happened in the thymus under the same experimental conditions, especially paying attention to mDCs (TCs), Thetis cells, Mimetic cells, and homing T cells in the Thymus, the medulla of the thymus could be the source of these pTregs.

### TGF-beta Signaling in the Development of CD8 Treg and CD4 Treg

It was well characterized that TGF-beta signaling plays a critical role in the development of CD4 Tregs by Chen’s group [11], which should be the first signal when self-reactive CD4 T cells meet tissue-specific antigens presented by mTECs in the Medulla zone. The activation of CD4 T cells by mTECs likely triggers the expression of TGF-beta receptors on the surface, which could sense the TGF-beta in the medulla zone and trigger the differentiation of self-reactive CD4 T cells into CD4 Tregs. However, the exact underlying mechanism of transcriptional expression of TGF-beta receptors has not been well explored so far. There is no report, including the current report from Badr [1], on how TGF-beta reshapes the self-reactive CD8 T cells in the thymus, and most likely in the Medulla zone as well.

### FOXO1 or FOXP1 and FOXP3 in the Development of CD8 Treg and CD4 Treg

It was well characterized that TGF-beta triggers the expression of FOXP3 in the development of CD4 Tregs in the medulla zone by Chen’s group [11]. It has been assumed that there must exist a population of FOXP3-positive CD8 Tregs; however, it turned out not to be the case since there are very few FOXP3-positive CD8 T cells *in vivo*, suggesting that there may exist another CD8-specific FOXP3-like transcription factor that is induced by TGF-beta signaling upon activation of the TCR in thymus CD8 T cells. Badr reported that FOXO1 is highly expressed in the immature CD4/8 dull double-positive population at the positive-selection stage, which is originally repressed by Gfi1 [1]. However, FOXO1 appears to be expressed in all naïve T cells, and it has also been shown to control the expression of TGF-beta receptors after initial activation of T cells in the Medulla zone. It will be important to identify the transcription factor (maybe a FOXP3-like) responsible for the development of CD8 Tregs. Interestingly, a report from Conejo-Garcia’s group showed that TGF-beta up-regulated FOXP1 and mediated the suppression of the antitumor effect of CD8 T cells, while the expression of FOXP1 confers a regulatory or exhaustion property on the tumor-infiltrating CD8 T cells [19]. An earlier report showed that FOXP1 is essential for the development of quiescent naïve T cells (Treg populations?) [20]. Another group showed that FOXP1 expression and TGF-beta pathway activities in human CD8 T cells were elevated after co-culture with M2 inhibitory macrophages [21]. These limited but crucial data may suggest that the expression of FOXP1 is controlled by TGF-beta in CD8 Tregs, which acts like FOXP3 in CD4 Tregs, though additional evidence is needed to confirm this speculation.

### CD8 Tregs, PD-1^+^CD39^+^ CD8 T cells, and Tumor-Infiltrating CD8 T cells (TILs)

As mentioned above, FOXP1 and TGF-beta-coupled regulation in CD8 T cells play a suppressive role in TILs [19]. Furthermore, they found that the depletion of FOXP1 improved antitumor immunity in ovarian cancer and sarcoma models [19]. Another group also showed that the expression of FOXP1 was increased in a tumor-bearing mouse, and overexpression of miR-149–3p, which inhibits FOXP1 translation, counteracted CD8 T cell exhaustion [22]. Interestingly, a recent report from Dr. John Wherry’s group showed that a PD-1^+^CD39^+^ CD8 T cell population, which were termed terminally exhausted CD8 T cells, derived from healthy humans has a similar transcription profile to those tumor-infiltrating CD8 T cells derived from cancer patients though they showed lower expression of FOXP1 (which may suggest lacking TGF-beta activation under regular environment compared to a high concentration of TGF-beta in the Medullar zone of the Thymus) [23]. This PD-1^+^CD39^+^ CD8 T cell TIL population is enriched for tumor-reactive cells both in primary and metastatic tumors [24]. Dr. Howard Chang’s group showed that treatment with anti-PD-1 could recruit novel clones of PD-1^+^CD39^+^ CD8 T cells into the tumor regions [25]. Interestingly, CD39 combined with CD73 is a signature marker for CD4 Treg [26]. It suggests that these CD39-positive CD8 T cells may belong to the CD8 Treg family, and CD39 could be a signature marker for CD8 Tregs as well. Based on the above analysis, we may conclude that CD8 Tregs, PD-1^+^CD39^+^ terminally exhausted CD8 T cells, and tumor-infiltrating CD8 T cells could be the same family.

### IL-15Rα and CD25 in the Development and Long-Term Survival of CD8 Treg and CD4 Treg

The IL-2 receptor CD25 is the specific signature surface marker of CD4 Tregs, which was used to isolate CD4 Tregs for the first time by Sakaguchi [7]. Interestingly, IL-2 receptors work in three forms: low-affinity receptor (Kd of ~10^−8^ M, CD25 alone), intermediate affinity (Kd of ~10^−9^ M, CD122 and CD132 heterodimer), and high-affinity (Kd of ~10^−11^ M, CD25, CD122, and CD132 heterotrimer) [27]. The expression of the trimer complex on the surface of CD4 Tregs suggests that CD4 Tregs could survive in the periphery with a very low concentration of IL-2 (~10^−11^ M), while activated CD4 T cells with only CD25 and activated CD8 T cells with only CD122 and CD123 could not survive when the IL-2 concentration is low. By the same token, it is also a challenge for hypothetical CD8 Tregs with only CD122 and CD132 on their surface to survive. An underappreciated report by Dai showed that IL-15Rα was highly expressed in a population of immunosuppressive CD122+ CD8 T cells that prevented allograft rejection even better than that of CD4 Tregs [28]. This may be the first isolation of CD8 Tregs with positive CD122. Interestingly, the report showed that IL-15 but not IL-2 could trigger the proliferation of this population, while IL-15 does not have any effect on CD4 Tregs [28]. It was reported that the IL-15Rα forms a trimer with CD122 and CD132 that has a high affinity (Kd of ~10^−11^ M) to IL-15 [27], suggesting that CD8 Tregs could survive at extremely low concentrations of IL-15 *in vivo*. We speculate that the self-reactive CD8 T cell population with positive CD122 reported by Badr [1] has a high likelihood of having the IL-15Rα on its surface, which could be a signature marker for CD8 Tregs, just like CD25 as the signature marker for CD4 Tregs. Similar to FOXP3 controlling the transcriptional expression of CD25, another FOX family member, FOXP1, may control IL-15Rα transcription in CD8 Tregs.

### PD-1 and CTLA4 are Specific for CD8 Treg and CD4 Treg Respectively

Besides the marker CD25 on the surface of CD4 Tregs, the inhibitory receptor CTLA4 is another signature marker on the surface of CD4 Tregs [29]. It is also a well-established immune checkpoint inhibitor target for CD4 T cells in various cancers. An excellent report by Walker’s group showed that CTLA4 functions by internalizing its ligands CD80 and CD86 [29], which are also ligands of CD28 that are essential for CD4 T cell survival. Another excellent report by Oxenius’ group [30] showed that deletion of PD-1 triggers the fatal pathology of mice due to over-reactive cell deaths induced by viral infections [30]. One explanation by the authors is that cytotoxic CD8 T cells could be overreactive to pathogen-infected host cells, which trigger massive cell death *in vivo* [30]. Badr’s report showed that CD8 Tregs gain tolerance through the expression of PD-1 on their surface [1]. This result suggested that the massive cell death observed in PD-1-deficient mice could be caused by the failure to establish CD8 Tregs, which tips the balance and causes the over-reaction of self-reactive CTLs in the event of viral infection.

### Both CD8 Treg and CD4 Treg Produce IL-10

Dai’s report showed that CD8^+^CD122^+^ Tregs have a more potent suppressive function against allograft rejection than CD4^+^CD25^+^ Tregs [28]. It showed that IL-15 could stimulate the expression of IL-10, a signature repressive cytokine, from the CD8 Tregs but not CD4 Tregs [28]. On the other hand, it is known that IL-2 could trigger the expression of IL-10 in CD4 Tregs but not CD8 Tregs. At the same time, it seems that both CD4 and CD8 Tregs could express TGF-beta and possibly other repressive cytokines when activated to shut down anti-self immune responses.

### Conclusion and Perspective

Based on the above analysis, we may finally draw a clearer picture of both regulatory T cell families, which include CD4 Treg and CD8 Treg (Figure 1). They share some common properties but have significantly different features. Overall, the reports by Badr [1], Dai [28], and others may allow us to finally define the CD8 Treg family, which is a key missing piece of the puzzle in the immune tolerance field. Most importantly, the dominant and parallel roles of CTLA4 and PD-1 in the two regulatory families explain why checkpoint inhibitors developed from these two targets may have dramatically different outcomes and toxicities in cancer patients. Recently, we proposed that the formation of memory T cells needs a two-step activation process for both memory CD4 T cells and memory CD8 T cells in the germinal centers [31]. It may also be true for both CD4 Tregs and CD8 Tregs; there is a need for a two-step activation process for the long-lived (lifetime) regulatory T cell population, just like memory T cell population [31]: activation in the thymus cortex zone during the positive selection process and activation in the thymus medulla zone during the negative selection process.

## Figures and Tables

**Figure 1.**
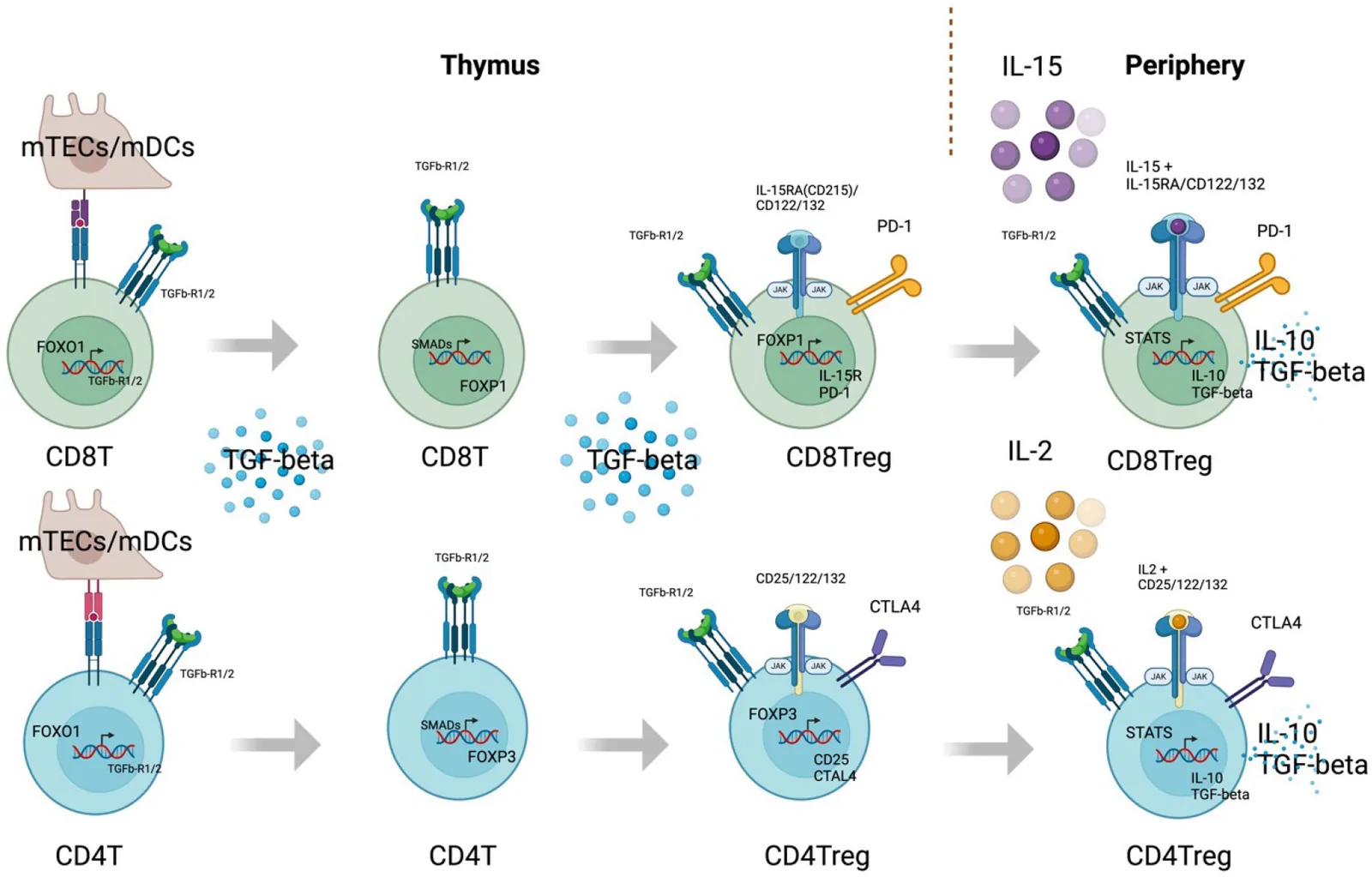
The development of CD4 and CD8 regulatory T cells.
